# Effectiveness of a Six-Week Multimodal Physiotherapy Program on the Interconnected Nature of Forward Head Posture, Vertigo, and Neck Pain

**DOI:** 10.7759/cureus.65038

**Published:** 2024-07-21

**Authors:** Mayuri R Zoting, Shubhangi Patil

**Affiliations:** 1 Community Health Physiotherapy, Ravi Nair Physiotherapy College, Datta Meghe Institute of Higher Education and Research, Wardha, IND

**Keywords:** physical therapy, gaze stabilization exercises, neck pain, vertigo, forward head posture

## Abstract

The forward head posture (FHP) is characterized by the head tilting forward compared to the shoulders, resulting in pressure on the neck and surrounding muscles, which may lead to chronic neck pain. The study focuses on a 47-year-old female patient with FHP experiencing symptoms such as dizziness and neck discomfort and emphasizes the importance of various treatment options. After six weeks of personalized training designed to improve posture and alignment, the patient's neck pain improved significantly. Evaluations using different assessments showed significant improvements in pain intensity, head and neck alignment, neck pain and function, severity of dizziness, and neck mobility. This case report discusses the role of physical therapy in treating muscle and balance problems to alleviate symptoms of various health conditions and shows lasting positive effects. It emphasizes the interconnection of conditions such as pain and dizziness and their impact on overall recovery and health. The physiotherapy approach aimed to improve patient outcomes and functional abilities by addressing muscular-skeletal and vestibular problems. This study highlights the complex relationship between FHP, vertigo, and neck pain.

## Introduction

Forward head posture (FHP) is caused by poor posture associated with forward shoulder posture and increased thoracic spine kyphosis [1]. Poor posture is related to alterations in the scapula's position regarding kinematics and muscle function [1,2]. The upper trapezius, forward along with a variety of other lower neck muscles such as the splenius, suboccipital, semispinalis, sternocleidomastoids, and levator scapulae, is the obstacle that leads to increased blood pressure in the upper cervical vertebrae through linear head placement [2]. FHP is a deviation from the normal posture because the head, in this case, leans too far forward from the shoulders due to rounding of the back and leaning of the shoulders. As a result, it can cause neck pain. If a person is involved in such a position for a prolonged duration, it is called forward head rounded shoulder posture (FHRSP). FHRSP essentially changes the movement of the muscle and the cervical spine alignment, thus increasing the amount of tension on the shoulders, causing pain, and worsening cervical spine function [3]. Hence, enhancing the FHRSP can help decrease shoulder tension [4]. Besides this, the so-called “forward head posture” may also modify body mechanics and can even disturb the vestibular system causing vertigo.

Multiple angles are utilized in FHP measurements, including craniovertebral angle (CVA), neck tilt angle, and head tilt angle [4]. FHP results in added pressure on the bones and muscles in the cervical spine, changes the muscle length and tension interaction in the upper cervical spine, limits head and neck movement, and hinders the neck's ability to detect its position in space [5]. When the head is pushed further forward, sagittal movement is increased in the upper spine's front vertebrae compared to the lower cervical spine [6]. Sensory stability and orientation depend on the combination of symmetrical input from the vestibular organs, visual and auditory signals, and proprioception in the nervous system [7]. Imbalance in sensory organs or afferent input can lead to dizziness and balance disorders [8]. Feeling lightheaded is prevalent among patients seeking medical care at the center. In the US adult population, the number of people experiencing vertigo within the past year was 8.4% [9]. While patients with vertigo usually receive a single diagnosis, about 3.7% of them may have more than one. Benign paroxysmal positional vertigo (BPPV), vestibular neuritis, and Meniere's disease are the diagnoses that are most commonly observed [10]. Cervicogenic dizziness (CGD) results from cervical spine trauma, inflammation, degeneration, or mechanical issues [11].

Numerous clinicians strongly believe in the correlation between vertigo and inadequate blood flow in the vertebral artery [8-12]. Individuals experiencing compression of the vertebrobasilar system frequently describe symptoms such as dizziness, vertigo, headaches, vomiting, double vision, lack of coordination, feeling off-balance, and weakness in bilateral limbs [13]. Assessment techniques like the vertebrobasilar insufficiency test and transcranial Doppler ultrasound are commonly accepted for identifying dizziness caused by vertebral artery insufficiency [14]. Changes in the upper neck region may be more strongly linked to feelings of dizziness than changes in the lower neck region [11]. Most of the movement in bending, stretching, or twisting of the neck happens in the upper part of the neck, specifically in the joints between the skull and the first two vertebrae [15]. Half of the cervical proprioceptors can be found in the joint capsules of C1-C3 [16]. The cervical spine's joint capsules play a vital role in proprioception via mechanoreceptors [17]. These sensory receptors are found in high numbers in the γ-muscle spindles situated deeply within the upper cervical muscles [18-19]. Mechanoreceptors found in the upper cervical spine are probably involved in regulating the activity of sensory nerves in the cervical spine [20]. Hence, a malfunction in the upper cervical area can change spatial orientation and result in feelings of dizziness and instability [11]. In this case report, a patient with FHP presented with symptoms of dizziness and neck discomfort. This report shows the physical therapy protocol for patients, which can improve the functional quality of life and intercorrelation with symptoms.

## Case presentation

Patient information

A 47-year-old woman presented to the physiotherapy department complaining of severe neck discomfort that had persisted for three months. She was uncertain of the cause, but the pain was deep and pulsating, making it hard to turn her head. In addition, she also had continuous and constant irritation in both hands without tingling or numbness. She rated the pain during relaxation times as six out of 10 on a pain-rating scale. Nevertheless, it hindered her from engaging in some of her daily routines, although some rest enabled her to recover temporarily. The patient mentioned that she experienced neck pain, migraines, and bouts of dizziness about two to three times. She found relief only when she laid on her back. She also noticed that the pain worsened when she moved her head, especially to the right side. The patient found relief from her discomfort by using a short heat pack and pain relievers. She reported that her neck pain was causing difficulty in performing household tasks, lifting items, cooking, bending down, and reaching overhead. Furthermore, she admitted that her work capacity had diminished due to this pain. She had a four-year history of hypertension and was on medication, taking metosartan LN 50 mg, amlodipine 10 mg, metoprolol, and telmisartan 40 mg once a day. She had no history of asthma or tuberculosis, and her BMI was 26.8 kg/m^2^. 

Physical examination

**Figure 1:**
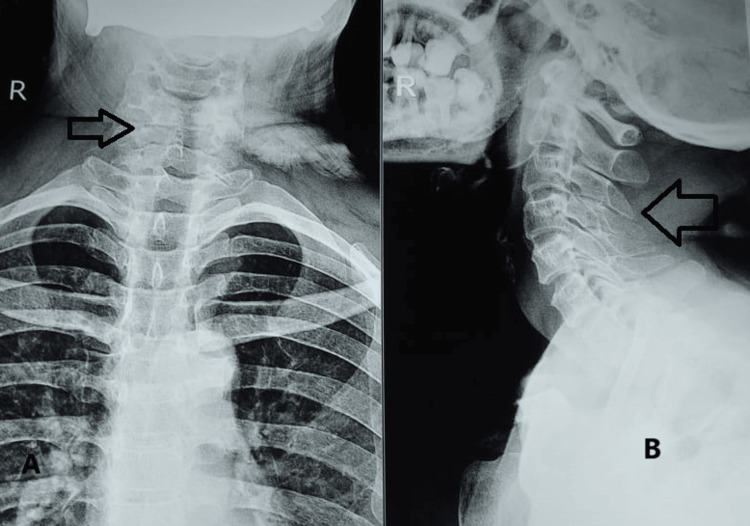
X-ray of the cervical spine in the anteroposterior (A) and lateral views (B) Evidence of a decreased gap between C6 and C7 as well as signs of wear and tear were observed in both A and B as shown by the arrows.

Forward head translation and rounded shoulders were observed during visual postural analysis. During the examination, limitations were observed in the movement of the neck, including extension, side bending on both sides, and rotation. During the muscle strength test, the cervical flexors scored 4/5, the extensors -4/5, the bilateral side flexors -4/5, and the rotators 4/5. The cervical vertebral angle (CVA) was found to be 44 degrees, and specific assessments showed positive results for the Spurling test, craniocervical flexion test, vertebral artery test, and transverse ligament stress test. A cervical spine X-ray investigation was performed with both anterior-posterior and lateral views, as shown in Figure 1.

Physiotherapy management

**Figure 2:**
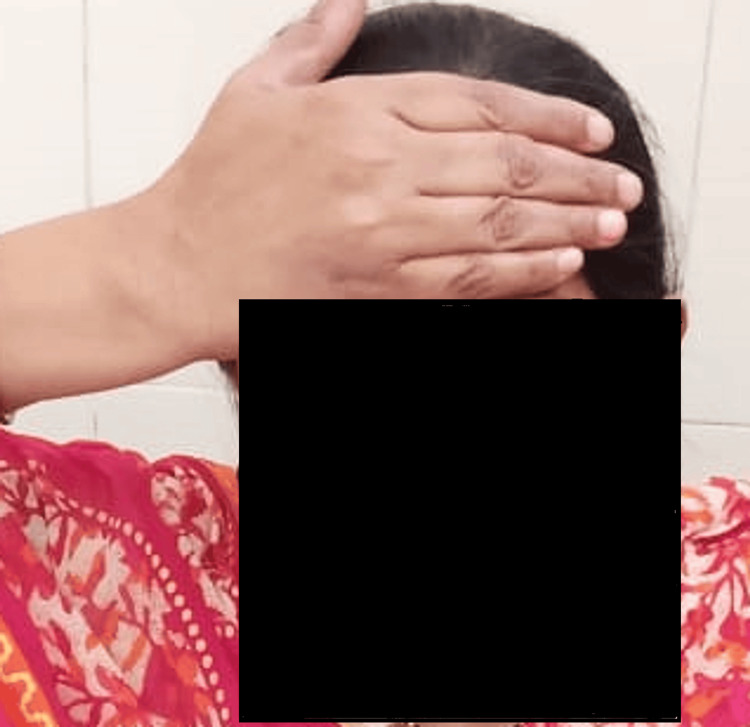
Patient performing isometric exercises for forward head posture for six weeks

The protocol for various exercises was given for six weeks; the physiotherapy management included postural correction exercises, isometrics of the cervical spine, scapular stabilization exercises, etc., as described in Table 1. The patient performed isometric exercises, as shown in Figure 2.

**Table 1:** The structured protocol of forward head posture for six weeks

Various exercises for the cervical spine	Exercise protocol	Repetition
Postural correction exercise	(1) Chin tucks in supine, wall chin tucks [1], (2) supine neck flexion, and (3) scapular protraction	Two sets lasting 30 to 40 seconds each.
Strengthening exercise for cervical spine	(1) Lateral neck flexion with depressed shoulder and (2) isometric neck exercises	Perform 2 sets of 5-8 repetitions, holding each rep for 3 seconds.
Scapular stabilization exercises	(1) Supine Y's, T's, W's and prone Y's, T's, W's in the extended position [21]; (2) wall slide; (3) doorway pectoralis stretch, doorway stretches, puppy dog stretch, and cat and cow yoga [21]	Perform 2 series of 5-10 repetitions each, holding for 5 seconds. 3 sets,10 seconds hold. 3 sets,30-60 seconds hold. 2-3 sets, 30-60 seconds hold. 2-3 sets, 30 seconds. 2 sets, 5 repetitions, hold 3 seconds at each position.
Vestibular rehabilitation	Gaze stabilizing exercises and Epley’s maneuver [22]	2 sets, 30-40 seconds, 5 repetitions
Manual therapy	Cervical traction and spinal manipulation [23]	5 repetitions, 2 seconds hold

Outcome measures

After six weeks of therapy, the patient experienced a significant reduction in neck pain and a decrease in both the occurrence and severity of vertigo episodes. Assessments were carried out before and after the treatment with objective measures such as the numeric pain rating scale (NPRS)/visual analog scale (VAS), CVA, cervical range of motion (ROM), neck disability index (NDI), and visual vertigo analog scale, as shown in Table 2.

**Table 2:** The pre- and post-outcome measures taken for six weeks NDI: Neck disability index; CVA: Craniovertebral angle; VAS: Visual analog scale; VVAS: Visual vertigo analog scale; ROM: Range of motion.

Outcomes	Pre-treatment	Post-treatment
VAS	6/10	2/10
CVA	60 degrees	50 degrees
NDI	30	25
VVAS	67/90	62/90

The pre- and post-outcome measures in six weeks for cervical range of motion are described in Table 3.

**Table 3:** The pre- and post-cervical range of motion taken for six weeks

Cervical active range of motion (CROM)	Active range of motion (Pre-ROM)	Active range of motion (Post-ROM)
Cervical flexion	65 degrees	75 degrees
Cervical extension	50 degrees	60 degrees
Left cervical rotation	70 degrees	80 degrees
Right cervical rotation	75 degrees	82 degrees
Left cervical lateral flexion	30 degrees	35 degrees
Right cervical lateral flexion	20 degrees	25 degrees

## Discussion

FHP is often associated with neck pain as the head is positioned in a forward manner on the neck. It has been suggested that this misalignment could result in increased pressure on the cervical components located at the back [24], impact the relationship between length and tension in the neck muscles [25,26], raise the level of muscular activity [27], and disrupt neck position sense [28]. Long-term FHP has been associated with a range of health issues, such as neck pain, headaches, cervical disc injuries, degeneration of the cervical spine, decreased lung function, and temporomandibular joint disorders [29]. A study found that FHP is a common postural anomaly. Scapular stabilization exercises (SSE) can help bring the thoracic cage and head back to a neutral position. A study involving 60 participants between the ages of 20 and 35 found that combining SSE with PCE was more effective in treating individuals with FHP than solely relying on PCEs. The study found that both SSE and PCE improved the CVA and pressure pain threshold while decreasing muscle activity and disability. Scapular stability had a more significant effect on these factors when compared to exercises focusing solely on correcting posture [11-14]. We utilized the identical procedure described earlier in these investigations. Before the exercises, the VAS score was 6/10, but after doing the postural correction exercises, the VAS score improved to 2/10. The patient received a six-week treatment including postural correction, stabilization exercises, and gaze stabilization exercises.

The results indicate that the VAS and NDI scores decreased after the treatment compared to the initial scores. In 2022, Arif et al. researched the impact of cervical stabilization exercises on long-term neck pain in individuals with FHP. Twenty people engaged in cervical stabilization exercises three times per week for four weeks along with traditional treatments, while another group of 20 people only received traditional treatment such as a heating pad, TENS, and cervical isometric exercises. The findings indicated that utilizing both cervical stabilization exercises and isometric exercises was more successful in decreasing CVA, pain, and neck disability compared to traditional treatment [30]. In 2021, Kim et al. researched the impacts of manual therapy. Eleven people underwent 12 therapy sessions focusing on joint mobilization in the cervicothoracic junction and upper cervical spine over four weeks. Both treatments improved the pain, dysfunction, and muscle activity of individuals with FHP. Notable enhancements in CVA and ROM of neck extension were observed by targeting the region where the neck connects to the upper back rather than focusing on the upper neck. Manual therapy was recommended as a beneficial option for enhancing neck mobility and posture during the study [31].

Dizziness might be caused by problems in the neck muscles from abnormal head position causing an accumulation of abnormal stimuli [32]. More specifically, issues in the upper neck area may have a stronger connection to vertigo than issues in the lower neck area [11]. This FHP will place greater strain on the neck and shoulder muscles and may even constrict blood flow to the brain. The alteration in blood flow throughout the body may therefore cause symptoms of vertigo such as dizziness and imbalance. In addition, misalignment of the head and neck causes dysfunction in the vestibular system that provides the brain with information that is essential for navigation and balance. The FHP can be controlled, and the intensity of dizziness or vertigo symptoms can be reduced by exercises and correcting posture [33]. However, some research suggests that not everyone benefits from techniques like the Epley maneuver when treating vertigo. In addition to this, both gaze stabilization exercises and the Epley maneuver have been found helpful in decreasing vertiginous attacks.

According to a study conducted by Cho et al., thoracic-spinal manipulation had better results in terms of forward head correction than cervical mobility [23]. After a six-week follow-up, the NPRS scores for NDI improved following the fourth week, with 68.8% of subjects showing a great response change (GRC) recorded at +4 and above among those exposed only to the thoracic spine [17]. The exercise includes PCE and SSE. The outcome measures taken pre- and post-exercise showed decreasing CVA, NDI, VAS, vertigo visual analog scale (VVAS), and cervical range of motion (CROM) scores. The patient's dizziness, as measured by the VVAS, decreased after the treatment. Before treatment, the score was 67 out of a possible 90. After treatment, the score improved to 62 out of 90. This study showed a decrease in the post scores through various measurements such as VAS, cervical ROM, NDI, CVA, and VVAS. This study highlights the links between FHP, vertigo, and neck pain. The unique physical therapy management mainly tackles the components of the musculoskeletal and vestibular system. Treatment that provides lasting benefits and the factors that influence these illnesses should be studied in the future.

## Conclusions

A combination of various physical therapy interventions was needed to treat FHP, vertigo, and neck pain. This case report states that targeting various musculoskeletal and vestibular issues with physiotherapy management provides superior outcomes and enhanced patient functionality.
